# Use of the Janssen (Johnson & Johnson) COVID-19 Vaccine: Updated Interim Recommendations from the Advisory Committee on Immunization Practices — United States, December 2021

**DOI:** 10.15585/mmwr.mm7103a4

**Published:** 2022-01-21

**Authors:** Sara E. Oliver, Megan Wallace, Isaac See, Sarah Mbaeyi, Monica Godfrey, Stephen C. Hadler, Tara C. Jatlaoui, Evelyn Twentyman, Michelle M. Hughes, Agam K. Rao, Anthony Fiore, John R. Su, Karen R. Broder, Tom Shimabukuro, Allison Lale, David K. Shay, Lauri E. Markowitz, Melinda Wharton, Beth P. Bell, Oliver Brooks, Veronica McNally, Grace M. Lee, H. Keipp Talbot, Matthew F. Daley

**Affiliations:** ^1^CDC COVID-19 Emergency Response Team; ^2^University of Washington, Seattle, Washington; ^3^Watts HealthCare Corporation, Los Angeles, California; ^4^Franny Strong Foundation, West Bloomfield, Michigan; ^5^Stanford University School of Medicine, Stanford, California; ^6^Vanderbilt University Medical Center, Nashville, Tennessee; ^7^Institute for Health Research, Kaiser Permanente Colorado, Denver, Colorado.

On February 27, 2021, the Food and Drug Administration (FDA) issued an Emergency Use Authorization (EUA) for the adenovirus-vectored COVID-19 vaccine (Janssen Biotech, Inc., a Janssen Pharmaceutical company, Johnson & Johnson), and on February 28, 2021, the Advisory Committee on Immunization Practices (ACIP) issued an interim recommendation for its use as a single-dose primary vaccination in persons aged ≥18 years (*1*,*2*). On April 13, 2021, CDC and FDA recommended a pause in the use of Janssen COVID-19 vaccine after reports of thrombosis with thrombocytopenia syndrome (TTS), a rare condition characterized by low platelets and thrombosis, including at unusual sites such as the cerebral venous sinus (cerebral venous sinus thrombosis [CVST]), after receipt of the vaccine.* ACIP rapidly convened two emergency meetings to review reported cases of TTS, and 10 days after the pause commenced, ACIP reaffirmed its interim recommendation for use of the Janssen COVID-19 vaccine in persons aged ≥18 years, but included a warning regarding rare clotting events after vaccination, primarily among women aged 18–49 years (*3*). In July, after review of an updated benefit-risk assessment accounting for risks of Guillain-Barré syndrome (GBS) and TTS, ACIP concluded that benefits of vaccination with Janssen COVID-19 vaccine outweighed risks. Through ongoing safety surveillance and review of reports from the Vaccine Adverse Event Reporting System (VAERS), additional cases of TTS after receipt of Janssen COVID-19 vaccine, including deaths, were identified. On December 16, 2021, ACIP held an emergency meeting to review updated data on TTS and an updated benefit-risk assessment. At that meeting, ACIP made a recommendation for preferential use of mRNA COVID-19 vaccines over the Janssen COVID-19 vaccine, including both primary and booster doses administered to prevent COVID-19, for all persons aged ≥18 years. The Janssen COVID-19 vaccine may be considered in some situations, including for persons with a contraindication to receipt of mRNA COVID-19 vaccines.

Since June 2020, ACIP has convened 23 public meetings to review data on the epidemiology of COVID-19 and the use of COVID-19 vaccines, including nine during which Janssen COVID-19 vaccine-related data were reviewed. The ACIP COVID-19 Vaccines Work Group, comprising experts in infectious diseases, vaccinology, vaccine safety, public health, and ethics, has held weekly meetings to review COVID-19 surveillance data, evidence for vaccine efficacy and safety, and implementation considerations for COVID-19 vaccines. In addition, the COVID-19 Vaccines Safety Technical Work Group (VaST), consisting of independent vaccine safety experts and established to provide expert consultation on COVID-19 vaccine safety issues, has reviewed safety data from the COVID-19 vaccination program during weekly meetings. After TTS was first identified in the United States in April 2021, a benefit-risk assessment for the use of the Janssen COVID-19 vaccine was presented to ACIP using an adapted Evidence to Recommendations (EtR) framework.^†^ In the setting of limited COVID-19 vaccine supply in the United States at that time, ACIP reaffirmed its interim recommendations for the use of the Janssen COVID-19 vaccine in persons aged ≥18 years under FDA’s EUA, which was updated to include a warning that rare clotting events might occur after vaccination, primarily among women aged 18–49 years (*3*). Updates to the benefit-risk assessment were also reviewed by ACIP in June 2021, after an increased risk for myocarditis, particularly in males aged 12–29 years, was observed after receipt of mRNA COVID-19 vaccines; and again, in July 2021, after an increased number of cases of GBS were identified following administration of Janssen COVID-19 vaccine (*4*,*5*). After each review, ACIP determined that the benefits of COVID-19 vaccination in preventing COVID-19 morbidity and associated mortality outweighed the risks for these rare, but serious adverse events; however, the balance of benefits and risks varied by age and sex. Ongoing postauthorization safety surveillance identified additional TTS cases and associated deaths after Janssen COVID-19 vaccination, and updated safety data were reviewed by VaST in December 2021. The COVID-19 Vaccines Work Group also reviewed an updated benefit-risk assessment of COVID-19 vaccines in the setting of new safety findings and sufficient COVID-19 vaccine supply in the United States. In addition, FDA updated the EUA fact sheets with additional TTS data in December 2021.^§^ A summary of the data reviewed and discussions from both VaST and the ACIP COVID-19 Vaccines Work Group were presented to ACIP during their emergency meeting on December 16, 2021.

TTS is a rare but potentially life-threatening syndrome associated with adenoviral-vectored COVID-19 vaccination that involves acute venous or arterial thrombosis and new onset thrombocytopenia (*6*). Based on the distinctive clinical and laboratory features of the syndrome, epidemiologic clustering in time after receipt of adenoviral-vectored COVID-19 vaccines, and plausible pathogenic mechanisms, the evidence supports a causal relationship between TTS and the Janssen COVID-19 vaccine (*6*). Potential adverse events, including cases of TTS, are reported to VAERS (*7*), the national passive vaccine safety monitoring system. Physicians at CDC and FDA confirmed whether each report met the CDC case definition for TTS^¶^ through medical record review, with input from Clinical Immunization Safety Assessment Project investigators,** including hematologists. A detailed review of TTS cases with vaccination occurring before August 31, 2021, including a description of rates, patient characteristics, and clinical course, was presented to ACIP and used in the benefit-risk analysis (*8*).

Overall, 54 cases of TTS were identified in persons who received the Janssen COVID-19 vaccine during March 2–August 31, 2021; 37 (69%) patients were women, 45 (83%) were White non-Hispanic persons, and the median age was 44.5 years (range = 18–70 years). Most patients (39; 72%) received the Janssen COVID-19 vaccine before the pause on April 13, 2021; 15 (28%) cases occurred in persons who were vaccinated after the pause was lifted on April 23, 2021. Whereas most (13 of 15; 87%) patients with TTS identified through April 2021 were women aged 18–49 years, approximately one half (26 of 54; 48%) of all patients with TTS after receipt of Janssen COVID-19 vaccine identified through August 2021 were women aged 18–49 years.

Approximately 14.1 million doses of the Janssen COVID-19 vaccine were administered in the United States through August 31, 2021, resulting in an overall TTS reporting rate of 3.83 cases per million doses administered. TTS rates were highest among women aged 30–39 years (10.6 per million doses) and 40–49 years (9.0 per million doses) (Table 1). Among persons who received primary Janssen COVID-19 vaccination by August 31, 2021, eight TTS deaths occurred^††^ (*8*). Six deaths occurred in women, and two in men. The overall reporting rate for TTS deaths was 0.6 per million Janssen COVID-19 vaccine doses administered; the highest rates were among women aged 30–39 years (1.9 per million doses) and 40–49 years (1.8 per million doses). Among the patients who died with TTS, six had a diagnosis of CVST, and two had clinical characteristics compatible with CVST; all eight had presenting features associated with poor short-term prognosis (e.g., cerebral hemorrhage, intracranial edema, and mass effect) (*9*). Although public health messaging concerning the risk associated with the Janssen COVID-19 vaccine and clinical guidance for management and treatment of TTS^§§^ was provided in April 2021, the proportion of deaths among reported TTS cases did not decline (five deaths among 39 [13%] TTS patients vaccinated before the pause and three deaths among 15 patients (20%) vaccinated after the pause), likely due to the rapidity of progression of severe CVST.

**TABLE 1:** Number of cases and deaths attributed to thrombosis with thrombocytopenia syndrome reported to the Vaccine Adverse Event Reporting System following administration of Janssen (Johnson & Johnson) COVID-19 vaccine, total Janssen COVID-19 vaccine doses administered, and reporting rate per million Janssen COVID-19 vaccine doses administered, by sex and age group — United States, March–August 2021

Sex/Age group, yrs	No. of TTS cases	No. of TTS deaths*	No. of Janssen COVID-19 vaccine doses administered	No. of TTS cases per million Janssen COVID-19 vaccine doses administered	No. of TTS deaths per million Janssen COVID-19 vaccine doses administered
**Women**					
**18–49**	26	4	3,235,530	8.0	1.2
18–29	5	0	1,089,649	4.6	0
30–39	11	2	1,037,386	10.6	1.9
40–49	10	2	1,108,495	9.0	1.8
**50–64**	9	2	2,002,984	4.5	1.0
**≥65**	2	0	1,096,923	1.8	0
**Men**					
**18–49**	12	2	4,402,102	2.7	0.5
18–29	3	1	1,565,212	1.9	0.6
30–39	3	0	1,443,900	2.1	0
40–49	6	1	1,392,990	4.3	0.7
**50–64**	5	0	2,338,263	2.1	0
**≥65**	0	0	1,004,285	0	0
**Total**	**54**	**8**	**14,080,087**	**3.8**	**0.6**

**Abbreviation:** TTS = thrombosis with thrombocytopenia syndrome.

* An additional death was reported in a woman aged 18–29 years who received the Janssen COVID-19 vaccine after August 31, 2021.

ACIP reviewed an updated benefit-risk assessment of COVID-19 vaccines to determine whether the interim recommendations for the use of the Janssen COVID-19 vaccine in the United States should be updated. This assessment considered 1) the incidence of TTS and case characteristics, 2) current COVID-19 epidemiology, 3) an individual benefit-risk analysis to quantify COVID-19 hospitalizations prevented by Janssen COVID-19 vaccination in the United States and possible vaccine-associated adverse events, 4) data from jurisdictional COVID-19 vaccination programs describing use of Janssen COVID-19 vaccine, and 5) administration of Janssen COVID-19 vaccine by age and sex. ACIP reviewed the benefits and risks of Janssen COVID-19 vaccination compared with no COVID-19 vaccination. Given the current widespread availability of mRNA COVID-19 vaccines, the analysis also included the differential benefits and risks of the Janssen COVID-19 vaccine compared with mRNA COVID-19 vaccines, using methods similar to those used previously.^¶¶^

The benefits of Janssen and mRNA COVID-19 vaccination over 180 days per million fully vaccinated persons*** aged ≥18 years were assessed, including 1) COVID-19 hospitalizations prevented, based on rates during the week ending November 13, 2021^†††^ and 2) age- and vaccine-specific vaccine effectiveness estimates from the Influenza and Other Viruses in the Acutely Ill (IVY) Network, a hospital-based platform that monitors effectiveness of influenza and COVID-19 vaccines.^§§§^ The risks assessed for Janssen COVID-19 vaccination were 1) updated TTS rates through August 31, 2021 and 2) GBS rates through June 30, 2021, reported previously to ACIP^¶¶¶^ (*5*). The risks for mRNA COVID-19 vaccination were based on myocarditis rates through October 6, 2021, previously reported to ACIP.**** Each benefit-risk assessment was stratified by sex and age group (18–49, 50–64, and ≥65 years). An additional aspect of the benefit-risk assessment included a review of the severity of vaccine-associated adverse events, including myocarditis, TTS, and GBS. Among 47 patients aged 12–29 years with myocarditis after mRNA COVID-19 vaccination and health care provider follow-up ≥3 months after diagnosis, preliminary data showed that 91% were deemed by their health care provider to have fully or probably recovered; further follow-up is ongoing.^††††^ Among fully reviewed deaths reported to VAERS, there have been no confirmed deaths due to myocarditis after mRNA COVID-19 vaccination. Among 130 patients with preliminary reports of GBS after Janssen COVID-19 vaccination through July 24, 2021, one (0.8%) patient died, and 18 (14%) had respiratory compromise or failure (*10*). Among 54 TTS cases after Janssen COVID-19 vaccination, eight (15%) patients died, and an additional nine (17%) required discharge to postacute care or a rehabilitation facility (*8*). The estimated benefits of the Janssen COVID-19 vaccine outweighed the risks when compared with no vaccine for all persons aged ≥18 years (Table 2). However, when compared with the benefit-risk balance for mRNA COVID-19 vaccines, the Janssen COVID-19 vaccine prevented fewer COVID-19 hospitalizations. In addition, potentially more severe, long-term health impacts from TTS and GBS after Janssen COVID-19 vaccination were noted, compared with the apparently less severe myocarditis-associated outcomes after receipt of mRNA COVID-19 vaccines.

**TABLE 2:** Estimated COVID-19 hospitalizations prevented during 180 days after administration of 1-dose Janssen (Johnson & Johnson) COVID-19 vaccine and 2-dose mRNA COVID-19 vaccine, number of cases of Guillain-Barré syndrome and thrombosis with thrombocytopenia syndrome cases expected per million Janssen vaccine doses administered and, number of myocarditis cases expected per million second mRNA vaccine doses administered, by sex and age group — United States, 2021

Vaccine/Sex/Age group, yrs	Benefits	Harms	
	No. of COVID-19 hospitalizations prevented*	No. of adverse events*	
**Janssen COVID-19 vaccine **		**No. of GBS cases**	**No. of TTS cases**
**Women**			
18–49	3,729	5	8
50–64	11,181	7	5
≥65	24,149	9	2
**Men**			
18–49	2,421	6	3
50–64	12,189	16	2
≥65	32,801	8	0
**mRNA COVID-19 vaccines (Pfizer-BioNTech or Moderna)**	No. of COVID-19 hospitalizations prevented*	**No. of myocarditis cases**	
**Women**			
18–49	4,700	2	
50–64	14,908	1	
≥65	27,962	0	
**Men**			
18–49	3,052	13	
50–64	16,251	1	
≥65	37,980	1	

**Abbreviations:** GBS = Guillain-Barré syndrome; TTS = thrombosis with thrombocytopenia syndrome.

*Per million doses administered

ACIP also reviewed population-level data, including current use of the Janssen COVID-19 vaccine. As of December 15, 2021, among approximately 488 million COVID-19 primary series doses and 56 million COVID-19 booster doses administered, only 17 million (3.5%) and 800,000 (1.6%), respectively, were Janssen COVID-19 vaccines.^§§§§^ According to 46 jurisdictional immunization programs that voluntarily completed an online form shared with jurisdictions during December 12–15, 2021, the Janssen COVID-19 vaccine was offered widely and was available among other COVID-19 vaccines to nearly all populations; however, in some transitional settings (e.g., correctional facilities, homeless shelters, or airports), it might have been the only vaccine offered.

Based on a comprehensive review of existing data, ACIP concluded that 1) because of both higher vaccine effectiveness of mRNA COVID-19 vaccines and more serious rare safety issues associated with the Janssen vaccine, the benefit-risk balance for mRNA COVID-19 vaccines is more favorable than that for Janssen COVID-19 vaccine, 2) a preferential recommendation for mRNA COVID-19 vaccines over the Janssen COVID-19 vaccine is warranted, 3) the benefits of Janssen COVID-19 vaccine continue to outweigh the risks of remaining unvaccinated, and 4) if Janssen COVID-19 vaccine is the only vaccine offered to some harder-to-reach populations, an inequitable distribution of risk for TTS and GBS might occur. These considerations were in the context of wide U.S. availability of mRNA COVID-19 vaccines. ACIP voted unanimously (15 to zero) for a recommendation for preferential use of mRNA COVID-19 vaccines over the Janssen COVID-19 vaccine for the prevention of COVID-19 for all persons aged ≥18 years.

ACIP members discussed concerns about the clinical severity of the very rare risk for TTS and GBS after Janssen COVID-19 vaccination. However, they highlighted that there might be some situations where Janssen COVID-19 vaccine could be offered, including to persons with a contraindication to mRNA COVID-19 vaccines (e.g., severe allergic reaction after a previous dose or to a component of an mRNA COVID-19 vaccine). In such situations, providing information concerning the risk for these rare but serious adverse events after Janssen COVID-19 vaccination will be critical to ensuring that vaccine recipients are making an informed decision. In addition, vaccine providers should be encouraged to start a 2-dose mRNA COVID-19 vaccine primary series, even if there is uncertainty about when or in what setting the patient will receive the second dose. Prioritizing availability of mRNA vaccines for use in hard-to-reach populations or in transitional settings and continued expansion of infrastructures affording mRNA vaccine access will be critical to ensuring equity in the opportunity to receive a preferentially recommended mRNA vaccine. Additional detailed clinical considerations for use of COVID-19 vaccines are available at https://www.cdc.gov/vaccines/covid-19/clinical-considerations/covid-19-vaccines-us.html.

CDC has updated patient education and communication materials reflecting the preferential recommendation for mRNA COVID-19 vaccines^¶¶¶¶^; timely updates of these materials are important to ensure that vaccine providers are aware of updated COVID-19 vaccine recommendations, that Janssen COVID-19 vaccine recipients are aware of these risks, and that they know to seek care if they experience concerning symptoms. CDC and FDA will continue to closely monitor reports of serious adverse events after both mRNA and Janssen COVID-19 vaccines and will present any additional data to ACIP for consideration. As demonstrated at the December 16, 2021, ACIP meeting, the benefit-risk analyses and ACIP recommendations for COVID-19 vaccines can be updated to reflect additional information as the COVID-19 pandemic evolves. All persons aged ≥5 years are recommended to receive a COVID-19 primary series vaccination with a preferred mRNA COVID-19 vaccine, and an mRNA COVID-19 booster dose, if eligible, particularly given the recent emergence of the highly transmissible B.1.1.529 (Omicron) variant.

## Reporting of Vaccine Adverse Events

FDA requires that immunization providers report vaccine administration errors, serious adverse events, cases of multisystem inflammatory syndrome, and cases of COVID-19 that result in hospitalization or death after administration of COVID-19 vaccine under an EUA.***** Adverse events that occur after receipt of any COVID-19 vaccine should be reported to VAERS (https://vaers.hhs.gov or 1-800-822-7967). Any person who administers or receives a COVID-19 vaccine is encouraged to report any clinically noteworthy adverse event, whether or not it is clear that a vaccine caused the adverse event. In addition, CDC has developed a new, voluntary smartphone-based online tool (v-safe) that uses text messaging and online surveys to provide near real-time health check-ins after receipt of a COVID-19 vaccine (https://www.cdc.gov/vsafe).

**Summary**

**What is already known about this topic?**

Cases of thrombosis with thrombocytopenia syndrome and Guillain-Barré syndrome have been reported after receipt of Janssen COVID-19 vaccine.

**What is added by this report?**

On December 16, 2021, after reviewing updated vaccine effectiveness and safety data, the Advisory Committee on Immunization Practices made a preferential recommendation for the use of mRNA COVID-19 vaccines over the Janssen adenoviral-vectored COVID-19 vaccine in all persons aged ≥18 years in the United States.

**What are the implications for public health practice?**

Pfizer-BioNTech or Moderna mRNA COVID-19 vaccines are preferred over the Janssen COVID-19 vaccine for primary and booster vaccination. The Janssen COVID-19 vaccine may be considered in some situations, including for persons with a contraindication to receipt of mRNA COVID-19 vaccines.

## Footnotes

*
https://emergency.cdc.gov/han/2021/han00442.asp

†
https://www.cdc.gov/vaccines/acip/recs/grade/downloads/acip-evidence-recs-framework.pdf

§
https://www.fda.gov/emergency-preparedness-and-response/coronavirus-disease-2019-covid-19/janssen-covid-19-vaccine#additional

¶ CDC case definition for TTS. Tier 1: thrombosis in an unusual location for a thrombus (i.e., cerebral vein, visceral artery or vein, extremity artery, central artery or vein) and new onset thrombocytopenia (i.e., platelet count <150,000 per microliter [*μ*L]) occurring any time after receipt of a COVID-19 vaccine. Tier 2: new-onset thrombocytopenia, thrombosis in an extremity vein or pulmonary artery in the absence of thrombosis at a Tier 1 location, and a positive antiplatelet factor (PF)4 antibody enzyme-linked immunosorbent assay test result or functional heparin-induced thrombocytopenia (HIT) platelet test occurring any time after receipt of a COVID-19 vaccine.

**
https://www.cdc.gov/vaccinesafety/ensuringsafety/monitoring/cisa/index.html

†† An additional death was reported in a woman aged 18–29 years who had received the Janssen COVID-19 vaccine after August 31, 2021.

§§
https://www.hematology.org/covid-19/vaccine-induced-immune-thrombotic-thrombocytopenia

¶¶
https://www.cdc.gov/vaccines/covid-19/info-by-product/janssen/risk-benefit-analysis.html

***
https://www.cdc.gov/coronavirus/2019-ncov/vaccines/fully-vaccinated.html

††† https://gis.cdc.gov/grasp/COVIDNet/COVID19_3.html. Data were used for the most recent week not subject to reporting delays before the ACIP meeting.

§§§ Vaccine effectiveness (VE) estimates from https://www.cdc.gov/mmwr/volumes/70/wr/mm7038e1.htm. Estimates are adjusted for continuous age in years, calendar date (biweekly), U.S. Department of Health and Human Services region, sex, and race/ethnicity. A combined VE estimate for both mRNA COVID-19 vaccines was used in the benefit-risk analysis: 18–49 years = 92%; 50–64 years = 92%; ≥65 years = 88%. VE estimates for Janssen COVID-19 vaccine: 18–49 years = 73%; 50–64 years = 69%; ≥65 years = 76%.

¶¶¶ Presumptive reports of GBS were not verified by medical record review. An interim analysis in the Vaccine Safety Datalink also found the risk for GBS (confirmed by medical record review) after Janssen COVID-19 vaccine was elevated during the 1–42 days after vaccination. https://www.medrxiv.org/content/10.1101/2021.12.03.21266419v1

****
https://www.cdc.gov/vaccines/acip/meetings/downloads/slides-2021-10-20-21/07-COVID-Su-508.pdf

††††
https://www.cdc.gov/vaccines/acip/meetings/downloads/slides-2021-11-2-3/04-COVID-Oster-508.pdf

§§§§
https://covid.cdc.gov/covid-data-tracker/#vaccinations_vacc-people-onedose-pop-5yr

¶¶¶¶
https://www.cdc.gov/vaccines/covid-19/info-by-product/janssen/index.html

*****
https://vaers.hhs.gov/reportevent.html
